# Observations on the Use of Cod Liver Oil, Matico, Chloroform, Tannin and Iron

**Published:** 1851-01

**Authors:** 


*Observations on the use of Cod Liver Oil, Matico, Chloroform, Tannin and Iron.* By N. W. CONDICT, M. D.

I take the liberty of submitting to the readers of your Gazette the following paper, which contains the result of my experience in the use of some of the drugs which have come into use in the course of the last few years. The principal articles are Cod Liver Oil, Chloroform, the Matico leaf, Tannin, Subnitrate of Bismuth and Citrate of Iron.

The Cod Liver Oil I have used in about thirty cases of tubercular consumption: in many cases of chronic Bronchitis; in some old cases of chronic rheumatism; in one very intractable case of Salt rheum eruption, covering nearly the whole body; in every variety of chronic inflammation of the mucous membrane; as a local application, combined with chalk or calamine powder, and in nearly every case with marked benefit. In consumption the patients who were too far gone to be cured, have in every instance been saved the aphthous sore mouth, and diarrhœa, and also the paroxysms of hectic fever, which almost universally attends the close of life in this disease.

Three or four of the cases of branchitis had gone through the whole round of topical applications, blisters, leeches, tartar emetic ointment, Dr. H———G———'s nitrate of silver swabs, &c., &c. But the oil, with the aid of cold water bandages to the neck, has left the patients in every case apparently cured. In some cases the stomach does not tolerate the oil, unless it be given after eating and then qualified by the elixir of Peruvian bark, or by wine, or some similar adjuvant. Where phthisis has advanced to its last stage, and the patient has hectic and diarrhœa, the bismuth in scruple doses, and the cold water bandages to the abdomen are of great service as adjuvants· If, at the same time, as is usual, the patients have much thirst, I encourage them to drink all the cold water they may incline to do, as it greatly aids, as I suppose, by its solvent power in the elimination of the hus, taken from the lungs into the circulation.

In the case of salt rheum, its value, as a topical application, was well proved by using it for two or three days on one side of the face, and not upon the other; upon one wrist, and not upon the other; and by varying it in this way many times over, and always with the same result.

Chloroform I have used in about fifty cases of labor, and always with the happiest effect—when used even while ergot is given, the operation of both goes on as if only one had been given. In Hysteria, or any other form of disease where convulsions supervene, its effect is instantaneous.

Taken in the incipient stages of inflammatory disease of any kind, by its sedative action upon the nervous system, and by withholding for the time it is being used, the supply of oxygenated blood from the tissues, it does much to smother the kindling up of the flame. It has been thought unsafe to be used in diseases of the lungs, but I used a pint of chloroform, in the case of a lady who died of consumption, in the course of the last twenty-four hours of her life, and by it saved her from all the ordinary suffering which marks that sad hour. In the convulsions of infants, I esteem it of more value than all the other remedies we have before known. In idiopathic neuralgia its use, if persevered in, will, I believe, always effect a cure. From experience in my own person, I can say that it has cured me of this disease, when morphine in doses of one grain repeated every hour, for six hours, had scarcely a perceptible effect. That disease so troublesome in children, Catarrh, gives way to two or three inhalations. To relax the muscles for reducing a fracture or dislocation, it acts to entire perfection—nor to do this need the patient be made at all insensible.

The only caution required is, not to give it upon a full stomach, nor exclude entirely the atmospheric air from passing into the lungs.

Of the Matico leaf and Tannin I will only say that they are most astringent.

The Matico leaf is powder, in tincture, in decoction, or in infusion is quite as potent as alum, Acetate of lead, or sulphate of Zinc. The Tannin is scarcely at all inferior to it.

The Sub-Nitrate of Bismuth, to check diarrhœa or acidity of stomach, no matter from what cause, is of very great value, but needs to be given in doses of a scruple to a drachm, and repeated as often as occurrences of symptoms calls for it.

The Citrate of Iron is an elegant preparation, and decidedly pleasant to the palate, and at least fully equal in virtue to either of the chaly beates.—*N. Y. Med. Gazette.*

